# Improvement of Laccase Production by *Thielavia terrestris* Co3Bag1. Enhancing the Bio-Catalytic Performance of the Native Thermophilic *Tt*LacA via Immobilization in Copper Alginate Gel Beads

**DOI:** 10.3390/jof9030308

**Published:** 2023-02-28

**Authors:** Marina Gutiérrez-Antón, Alejandro Santiago-Hernández, Johan Rodríguez-Mendoza, Claudia Cano-Ramírez, Ismael Bustos-Jaimes, Guillermo Aguilar-Osorio, Jorge E. Campos, María Eugenia Hidalgo-Lara

**Affiliations:** 1 Laboratory Ingeniería de Proteínas, Departamento de Biotecnología y Bioingeniería, CINVESTAV-IPN, Av. Instituto Politécnico Nacional No. 2508, Ciudad de México 07360, Mexico; 2 Laboratory Variación Biológica y Evolución, ENCB-IPN, Prol. De Carpio y Plan de Ayala s/n, Col. Santo Tomas, Ciudad de México 11340, Mexico; 3 Laboratory Fisicoquímica e Ingeniería de Proteínas, Departamento de Bioquímica, Facultad de Medicina UNAM, Ciudad Universitaria, Ciudad de México 04510, Mexico; 4 Grupo de Fisiología de Hongos, Departamento de Alimentos y Biotecnología, Facultad de Química UNAM, Ciudad Universitaria, Ciudad de México 04510, Mexico; 5 Laboratory Bioquímica Molecular, UBIPRO, FES Iztacala, UNAM. Av. de los Barrios No. 1, Los Reyes Iztacala, Tlalnepantla de Baz 54090, Mexico

**Keywords:** *Thielavia terrestris*, fungal laccase, alginate immobilization, thermophilic thermostable enzyme

## Abstract

A 32-fold increase in laccase activity production by the thermophilic biomass-degrading fungus *T. terrestris* Co3Bag1 was achieved when the microorganism was grown on a modified medium containing fructose, sodium nitrate, and copper. A 70 kDa laccase (*Tt*LacA), produced under the above conditions, was purified, immobilized in copper alginate gel beads, and characterized. *Tt*LacA, both free and immobilized enzymes, exhibited optimal activity at pH 3.0, at a temperature of 65 and 70 °C, respectively, although both displayed 70% of activity from 40 to 70 °C. Free and immobilized enzymes retained at least 80% of relative activity in the pH range from 3 to 4.6. Immobilized *Tt*LacA manifested a 2.3-fold higher thermal stability than the free form of the enzyme at 60 and 70 °C. Immobilized *Tt*LacA retained 95% initial activity for six consecutive reuse cycles at 60 °C, and also retained 86% of initial activity after 12 days of storage at 4 °C. Based on the biochemical features, thermophilic *Tt*LacA may be an efficient enzyme for dye decolorization and other industrial applications at high temperatures or acidic conditions. This work represents the first report about the immobilization and biochemical characterization of a thermophilic laccase from a member of the genus *Thielavia.*

## 1. Introduction

Laccases (*p*-diphenol oxidase, E.C. 1.10.3.2) are metalloproteins belonging to the family of blue multicopper oxidases (MCOs) and generally contain three cupredoxin-like domains [1]. The catalityc core integrates four copper atoms that depend on spectroscopic and electronic paramagnetic resonance (EPR) signals, and are classified as Type I (T1), paramagnetic “blue” copper; Type 2 (T2), paramagnetic “non-blue”; and Type 3 (T3), the diamagnetic spin-coupled copper-copper pairing [2]. T1 “blue” copper shows an intense electronic absorption at 610 nm by the covalent copper-cysteine bond that is responsible for the characteristic blue color, and it is where the oxidation of the reducing substrates (phenolic and non-phenolic compounds) occurs; T2 “non-blue” copper does not show absorption in the visible region but is EPR signal detectable; and T3 copper-copper shows absorption at 330 nm and absence of an EPR signal. One Type 2 copper and two Type 3 coppers conform to a tri-nuclear cluster and are connected to each other. The “four copper atoms” structure in the catalytic core is maintained through a highly conserved internal electron transfer Cys-His pathway, where one oxygen molecule is reduced to two molecules of water [2].

Catabolic processes carried out by laccase only require the presence of substrates and reduce molecular oxygen to water as a by-product, which has made this enzyme an ideal candidate for several biotechnological applications, e.g., the paper industry, in the decolorization of textile dyes, in the food industry as stabilizing agent and clarification, biofuels production, and pharmaceutical-cosmetic industries [3].

The production of large amounts of laccase has been needed for its use at an industrial scale under different strategies, mainly in search of new fungi species or modifying the nutritional and physiological conditions during the cultivation of a known species [4]. Laccase production is regulated by several conditions such as nutrients, the source and concentration of carbon, nitrogen [4,5], and aromatic compounds and metal ions used as inducers [6,7]. Cu^2+^ has been reported as the best cofactor of laccase and the inducer of this enzymatic activity in many fungi, including a recombinant laccase [8].

On the other hand, the advantages of laccase immobilization are the increase in enzymatic thermal stability, its resistance to chemical reagents, and its reusability [9]. Different enzyme immobilization techniques, respectively, enable physical or chemical interactions such as entrapment/encapsulation, adsorption, covalent binding, and self-immobilizations [9,10]. Alginate is a linear unbranched biopolymer with physical and chemical properties that allows it to form a polysaccharide matrix for the entrapment of either enzymes or whole cells [11]; in addition, enzyme entrapment in alginate gel beads is easy, it avoids the auto-proteolysis of proteases, is a GRAS (Generally Recognized as Safe) component, has a low cost, and is widely available [11].

Laccases are produced mainly by plants, fungi, bacteria [12], and algae [13]. Fungal laccases are primarily present in basidiomycetes, particularly white-rot fungi, the highest laccase producers [3]. Laccase production has been found in ascomycetes, although this issue has been studied much less in this kind of fungi. *T. terrestris* (*syn Thermothielavioides terrestris* [14]) is a soil-borne thermophilic ascomycete that can grow at a relatively low pH (pH 4.5) and elevated temperature (40–45 °C) [15]. Berka et al. [16] reported the genome sequence of *T. terrestris* NRRL 8126 and identified several enzymes and expression levels in the fungal secretomes. Recently, an analysis of the genome and transcriptome of *T. terrestris* LPH172 has been reported [17]. In particular, the thermophilic biomass-degrading fungus *T. terrestris* Co3Bag1 was isolated from sugarcane bagasse compost, and, to date, two enzymes have been reported, a β-1,4-xylanase *Tt*XynA and an exo-β-1,3-glucanase *Tt*Bgn31A [18,19]. However, there are few reports describing asco-laccases, and, to our knowledge, studies about the physiology of this fungus are limited. In this study, we focused on the production of laccases by *T. terrestris* Co3Bag1 under submerged fermentation containing different carbon and nitrogen sources and copper as an inducer of laccase activity. Additionally, one of the enzymes involved in the laccase activity produced by *T. terrestris* Co3Bag1 was purified and immobilized in copper alginate gel beads.

## 2. Materials and Methods

### 2.1. Microorganism and Culture Conditions

*T. terrestris* Co3Bag1 (CDBB-H-1938), was previously described [18], and maintained on potato dextrose agar (PDA) at 4 °C.

Spores of *T. terrestris* Co3Bag1 were obtained from the solid basal medium as previously described by Tien and Kirk [20] at 45 °C for 12–15 days. The fermentation medium used for laccase production was described by Zouari-Mechichi et al. [21]; the submerged fermentation was carried out at 45 °C in 250 mL Erlenmeyer flasks containing 100 mL of basal medium and was inoculated with 1 × 10^6^ spores. Cultures of *T. terrestris* Co3Bag1 were incubated at 45 °C on a rotary shaker (120 rpm). Every 24 h for 15 days, each flask was assayed for laccase activity and biomass. The determination of fungal biomass was established by dry weight and estimated in grams per liter (g/L); the culture of *T. terrestris* Co3Bag1 was filtered through pre-weighed Whatman No. 1 filter paper, then the filter´s paper with the retained biomass was dried in an oven set at 80 °C until a constant weight was obtained.

### 2.2. Effect of Carbon and Nitrogen Sources on Laccase Activity

The effect of carbon and nitrogen sources on the laccase production was carried out by single-factor experiments using the modified medium described by Zouari-Mechichi et al. [21]; CuSO_4_ was excluded from the trace elements solution unless otherwise stated. To test the impact of the carbon source on the output of laccase activity by *T. terrestris,* Co3Bag1 was evaluated by substituting glucose with fructose, maltose, or starch (10 g/L). To test the effect of the nitrogen source on the production of laccase, the activity was determined by replacing ammonium tartrate with peptone, yeast extract, ammonium sulfate, or sodium nitrate (2 g/L), which were evaluated individually using the best carbon source previously selected. Culture supernatant was collected to analyze laccase activity and protein content from the following day till the end of cultivation for 15 days. The effect of four media, with different carbon/nitrogen sources concentrations, on the production of laccase activity by *T. terrestris* Co3Bag1, was studied as follows: (1) HCLN (high carbon and low nitrogen) medium contained 20 g/L carbon and 2 g/L nitrogen; (2) LCLN (low carbon and low nitrogen) medium contained 5 g/L carbon and 2.0 g/L nitrogen; (3) HCHN (high carbon and high nitrogen) medium contained 20 g/L carbon and 5 g/L nitrogen; and (4) LCHN (low carbon and high nitrogen) medium contained 5 g/L carbon and 5 g/L nitrogen.

### 2.3. Effect of Copper Addition on Laccase Activity

To study the effect of the addition of copper on the production of laccase activity, CuSO_4_ was added at different final concentrations (0.2, 0.5, 1.0, 2.0, and 2.5 mM) to the culture of *T. terrestris* Co3Bag1 grown in each of the selected modified medium (HCLN, LCLN, HCHN, or LCHN) on day 0 and after the 4th and the 8th day of cultivation. Culture supernatant was collected to assay laccase activity and protein concentration at intervals of 24 h for 15 days.

### 2.4. Laccase Assay and Protein Determination

Laccase activity was determined by measuring the oxidation of 2,2’-azino-bis (3-ethylbenzothiazoline-6-sulfonic acid) (ABTS) (Sigma-Aldrich, St. Missouri, USA) as a laccase substrate using a molar extinction coefficient of ε = 36,000 M^−1^cm^−1^ [22]. The reaction mixture (1 mL) consisted of 0.1 mL enzyme-containing sample, 0.8 mL of 100 mM glycine-HCl buffer, pH 3.0, and 0.1 mL of 5 mM ABTS as the substrate. Assay mixtures were pre-incubated at 50 °C for 5 min before adding ABTS to initiate the reaction. The change in absorbance of the reaction mixture containing ABTS was monitored spectrophotometrically at 420 nm (A_420_) using the GENESYS 10 UV/Vis spectrophotometer (Thermo Fisher Scientific, Carlsbad, CA, USA). One unit of laccase activity (U) was defined as the amount of enzyme required to oxidize 1 μmol of ABTS per minute under the described conditions. Protein was determined by the method of Lowry et al. [23], using bovine serum albumin (Life Technologies, Carlsbad, CA, USA) as the standard. The results were reported as protein concentration in milligrams per milliliter (mg/mL).

### 2.5. Enzyme Purification

The purification process was carried out from the culture supernatant of *T. terrestris* Co3Bag1 by anion exchange chromatography. Fungal cultures were harvested and pooled after 8 to 10 days of cultivation. The mycelia were removed by filtration, then the culture supernatant was concentrated to 10 mL by ultrafiltration using a polyethersulfone membrane with a cutoff weight of 50,000 Da (Pall Corp, Port Washington, NY, USA) at 4 °C. The ultra-filtrate was dialyzed against Buffer A (50 mM histidine, 25 mM KCl, pH 6.0, 0.1 mM phenyl methyl sulfonyl fluoride (PMSF), and 5% (*v*/*v*) glycerol) for 12 h. Then, the dialyzed material was loaded onto a UNOsphere Q (Bio-Rad, Hercules, CA, USA) equilibrated column with Buffer A; then, the proteins that bound to the column were eluted by applying a linear gradient of KCl (0.025–1 M) in Buffer A at a constant flow rate of 2.0 mL/min. For further studies, fractions showing laccase activity were pooled and concentrated using a filtration device with a 10 kDa cutoff (Pall Corporation, NY, USA).

### 2.6. SDS-PAGE Analysis and Zymography

The molecular mass of the purified protein was estimated by SDS-PAGE using 10% polyacrylamide gel [24] and molecular weight (MW) markers as a reference (Bio-Rad, Hercules, CA, USA). Gels were recorded and analyzed using a gel documentation system (DigiDoc-It Imaging System, UVP). Zymogram analysis was carried out as previously described by Moin et al. [25], with some modifications. Protein samples collected on the higher activity days were separated in a 10% polyacrylamide gel at 37 °C for 30 min under non-denaturing conditions, i.e., without sodium dodecyl sulfate (SDS) and 2-mercaptoethanol.

Then, after electrophoresis, the gel was incubated at 50 °C for 10 min in 100 mM glycine-HCl buffer (pH 3.0), containing 5 mM of the substrate. Protein bands with activity laccase were visualized by developing a green and orange-colored band using ABTS and 2,6-dimethoxyphenol (2,6-DMP) (Sigma-Aldrich, St. Missouri, USA), respectively, as the substrate.

### 2.7. Immobilization of Laccase from T. terrestris Co3Bag1

Laccase was immobilized by entrapment in alginate gel beads under the conditions described by Sondhi et al. [26]. The alginate-enzyme mixture was prepared using purified laccase (100 U/L) and sodium alginate solution (3%, *w*/*v*) on a rotary shaker at 25 °C; the mixture was then dropped into 300 mM of CuSO_4_ solution. The resulting beads were left to be cold-hardened overnight at 4 °C. Then, copper alginate gel beads were filtered and washed several times with distilled water. The immobilization was monitored by detecting laccase activity in washed water. Immobilization efficiency was calculated using the following relationship [27]:

Immobilization efficiency (%) = (a_imm_/a_free_) × 100

where a_imm_ is the specific activity of the immobilized enzyme (U/mg) and a_free_ is the specific activity of the free enzyme (U/mg).

### 2.8. Biochemical Characterization of the Free and Immobilized Laccases

#### 2.8.1. Optimal pH and pH Stability

The optimal pH for immobilized laccase and the free form of the enzyme was determined at pH values ranging from 2.2 to 7.0 in 100 mM of different buffers using ABTS as the substrate: glycine-HCl buffer (pH 2.2–3.6) and citrate-phosphate buffer (pH 3.6–7). Reaction samples were incubated at 50 °C for 5 min. The pH stability of the free and immobilized enzyme forms was determined by pre-incubating the enzyme in the buffers, as mentioned above (pH values ranging from 2.2 to 7.0), at 25 °C for 50 min. Then, the remaining laccase activity was measured under standard conditions.

#### 2.8.2. Optimal Temperature and Thermal Stability

The effect of temperature on the laccase activity was evaluated at different temperatures ranging from 25 to 90 °C for 5 min, and at an optimal pH in the selected buffer. The thermal stability of free and immobilized laccase was determined by pre-incubating the free and immobilized enzyme forms at 60 and 70 °C, at optimal pH, from 2 to 4 h. The half-life (t_1/2_), corresponding to the time when 50% of the original activity is reached for each temperature, was calculated. The activities were calculated as a percent of relative activity regarding the highest activity, which was considered as 100%.

#### 2.8.3. Kinetic Parameters

Kinetic parameters *K*_m_ and *V*_max_ of free and immobilized laccase were determined by ABTS oxidation at different concentrations (0.05 to 5 mM) under optimal conditions for the activity of free (pH 3.0, 65 °C, and 5 min) and immobilized (pH 3.0, 70 °C, and 5 min) forms of the enzyme. The *K*_m_ and *V*_max_ values were calculated by the Lineweaver–Burk double reciprocal plot; then, the turnover number (*k*_cat_) was calculated using the ratio between the *V*_max_ and the concentration of the active enzyme. The catalytic efficiency was calculated using the ratio between *k*_cat_/*K*_m_.

#### 2.8.4. Effect of Metal Ions and Inhibitors on Free and Immobilized Laccase

The effect of several metal ions (Na^+^, Cu^2+^, Ca^2+^, Co^2+^, Zn^2+^, Mg^2+^, Hg^2+^, and Fe^2+^) and other compounds (ethylenediaminetetraacetic acid (EDTA) and NaN_3_) was evaluated by incubating free and immobilized laccase with each of the compounds at 1 and 10 mM final concentrations under optimal conditions. In the absence of metal ions or other compounds, the laccase activity was considered as 100% of relative activity.

### 2.9. Storage Stability and Reusability

Storage stability was evaluated by storing free and immobilized laccase preparations in a glycine-HCl buffer (100 mM, pH 3.0) at 4 °C for 24 days. Throughout, aliquot samples were collected and assayed every three days to determine laccase relative activity. The reusability of the immobilized enzyme was determined by assaying the laccase activity remaining after incubation at 60 °C, using ten copper alginate gel beads per cycle. Immobilized beads were recovered by filtration and washed thoroughly with distilled water and a glycine-HCl buffer (100 mM, pH 3.0). The procedure, as mentioned above, was repeated for eight consecutive cycles. Laccase activity in the first cycle was considered as 100% of relative activity.

### 2.10. Partial Amino Acid Sequencing and Sequence Analysis

Purified laccase was partially sequenced by tandem Mass Spectrometry (MS/MS) at the Laboratorio Universitario de Proteómica, Instituto de Biotecnología (IBT, UNAM). The amino acid sequence of peptides obtained was analyzed for similarity using Proteome Discoverer 1.4 (Thermo Scientific) (https://www.thermofisher.com/order/ accessed on 22 July 2017). The 3D crystal structure laccase from *Melanocarpus albomyces* MaLac1 (UniProtKB: Q70KY3, PDB code 3QPK) (http://www.rcsborg accessed on 23 July 2017) was used as a template in the ESPript program (http://espript.ibcp.fr/ESPript/ESPript/ accessed on 24 July 2017) [28] to determine the secondary structure elements of the purified laccase and other fungal laccases.

### 2.11. Phylogenetic Reconstruction

#### 2.11.1. Phylogenetic Relationships among the Phylum Ascomycota and Basidiomycota

The putative multicopper laccase phylogenetic analysis from *T. terrestris* NRRL 8126 (UniProtKB: G2R0D5) was made with other fungal laccases, including the two phyla Ascomycota and Basidiomycota. The amino acid sequences were obtained from the UniProtKB database (https://www.uniprot.org/help/sequences accessed on 18 August 2020). The multiple sequence alignment of the sequences was performed using MUSCLE with MEGA v 7.0.26 [29]. Phylogenetic reconstruction analysis was inferred using MRBAYES 3.2.6 statistical method based on the Poisson correction model [30], and implemented in Geneious 11 [29]; the phylogeny test used was the Bootstrap method with 500,000 bootstrap pseudo-replicates. The initial phylogenetic tree was obtained by applying Neighbor-Join and BioNJ algorithms using a JTT model and selecting the topology with a superior log-likelihood value. The amino acid sequence of laccase-4 from *Trametes versicolor* (UniProtKB: Q12719) was used as the out-group sequence for this analysis.

#### 2.11.2. Phylogenetic Relationships among the Phylum Ascomycota, Basidiomycota, and Magnoliophyta

Construction of the phylogenetic tree was performed using PhyML (http://www.atgc-montpellier.fr/phyml/ accessed on 26 September 2020) [31]. The closed multicopper laccase matching reference sequences were downloaded from GenBank (https://www.ncbi.nlm.nih.gov/genbank/ accessed on 26 September 2020) and aligned with the problem sequence from *T. terrestris* NRRL 8126 using default parameters in ClustalX [32]. The best nucleotide substitution model was estimated directly by the PhyML program based on the Akaike Information Criterion (AIC) [31]. The LG model (−lnL = 35,095.31, gamma shape parameter = 1.318, I = 0.005) was selected for the data set. The confidence test was estimated for each node via bootstrap analysis after 1000 pseudo-replicates. The *Gonoderma boninense* laccase 2 (UniProtKB: A0A5K1JZ75) was included as the out-group sequence in this analysis.

## 3. Results

### 3.1. Growth and Laccase Production Kinetic of T. terrestris Co3Bag1

**Figure 1:**
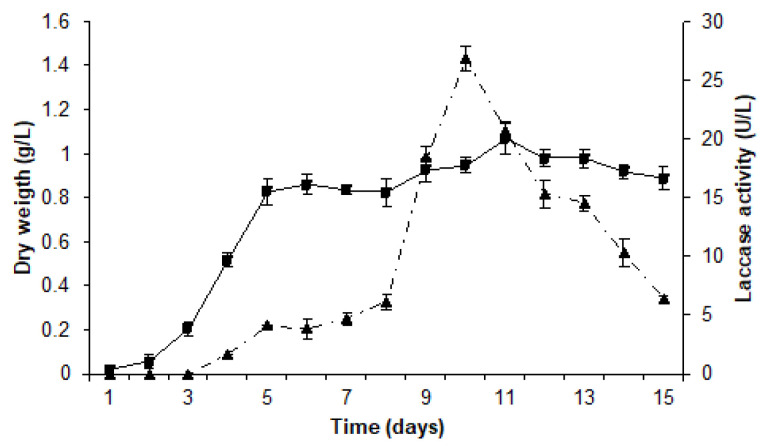
Growth and laccase production kinetics of *T. terrestris* Co3Bag1. Growth (■) and laccase activity (▲, dotted line) kinetics of *T. terrestris* Co3Bag1 in medium Zouari Mechichi et al. [21]. Dry cell weight monitored growth, and laccase activity was assayed using ABTS as substrate.

The time course kinetics of growth and laccase production was monitored in cultures of *T. terrestris* Co3Bag1. A gradual increase in laccase activity was registered as mycelia grew from day 1 to 8; a significant rapid increase was then registered from day 8 to 10; finally, post-day 10, a rapid decline was observed. These different patterns of change are correlated to logarithmic, stationary, and decline phases in the growth of the fungal culture. The greatest laccase activity (26.9 U/L) was reached after ten days of cultivation, with a final dry biomass accumulation of 0.95 g/L (Figure 1).

### 3.2. Effect of Carbon and Nitrogen Sources on Laccase Production

**Figure 2:**
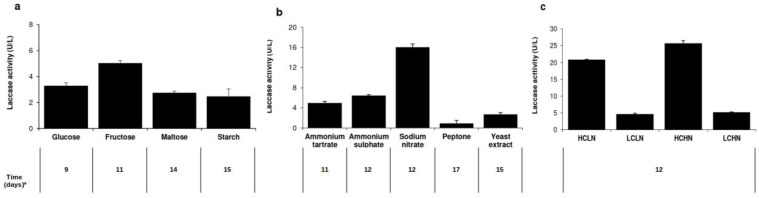
Effect of different carbon and nitrogen sources on the laccase production by *T. terrestris* Co3Bag1, (**a**), carbon sources (10 g/L); (**b**), nitrogen sources (2 g/L); (**c**), HCLN (high carbon, 20 g/L, and low nitrogen, 2 g/L); LCLN, (low carbon, 5 g/L, and low nitrogen, 2.0 g/L); HCHN, (high carbon, 20 g/L, and high nitrogen, 5 g/L); and LCHN (low carbon, 5g/L, and high nitrogen, 5g/L). * Time (days) corresponding to the day of maximal laccase activity recorded.

Firstly, we evaluated the influence of carbon sources (glucose, fructose, maltose, or starch, 10 g/L each) on laccase production by *T. terrestris* Co3Bag1, using (2 g/L) ammonium tartrate as the nitrogen source in all cases (Figure 2a). Laccase activity was observed on all carbon sources tested in this study; however, maximum laccase activity (5.03 U/L) was registered using fructose after 11 days of culture, which differs from laccase activity levels observed when *T. terrestris* Co3Bag1 was grown on maltose (2.76 U/L, day 14), starch (2.48 U/L, day 15), or glucose (3.3 U/L, day 9) as the carbon source. Secondly, in line with the analysis of the previously described data, fructose was designed as the best carbon source for the production of laccase activity by *T. terrestris* Co3Bag1 and used to assay the effect of the addition of different nitrogen sources (2 g/L each), both organic and inorganic, on the production of laccase activity by *T. terrestris* Co3Bag1 (Figure 2b). Maximum laccase activity (16.02 U/L, day 12) was registered using sodium nitrate, which is different from that recorded for ammonium sulfate (6.47 U/L, day 12), ammonium tartrate (4.97 U/L, day 11), yeast extract (2.69 U/L, day 15), or peptone (0.93 U/L, day 17) as the nitrogen source. All reported results correspond with the maximum laccase activity reached within the 17-day incubation period. Thus, fructose and sodium nitrate proved to be the best combination of carbon and nitrogen sources for *T. terrestris* Co3Bag1 to produce maximum laccase activity of 16.02 U/L after 12 days of incubation.

Then, once the best combination of carbon and nitrogen sources for laccase activity production by *T. terrestris* Co3Bag1 had been selected, we studied the effect of varying fructose and sodium nitrate concentrations on the 12th day of incubation for the production of laccase activity (Figure 2c). The greatest production of laccase activity by *T. terrestris* Co3Bag1 was registered in the HCHN medium with maximum laccase activity of 25.73 U/L, followed by the HCLN medium with maximum laccase activity of 20.85 U/L. Sodium nitrate concentration showed no significant influence on laccase activity; when the fungus was grown in LCLN or LCHN media, laccase production dropped drastically to 4.67 and 5.16 U/L, respectively. Thus, all data obtained indicate that HCHN formulation was the best medium for producing laccase activity by *T. terrestris* Co3Bag1 under the conditions tested. Laccase production by *T. terrestris* Co3Bag1, grown on medium HCHN, was 0.96-fold lower than the output obtained using the medium described by Zouari-Mechichi et al. [21]. Nevertheless, the HCHN medium was selected to study the effect of copper on laccase production by *T. terrestris* Co3Bag1 because the Zouari-Mechichi medium contains one carbon (glucose) and three different nitrogen sources (ammonium tartrate, peptone, and yeast extract).

### 3.3. The Effect of Copper on Laccase Production by T. terrestris Co3Bag1 Grown in HCHN Medium

**Figure 3:**
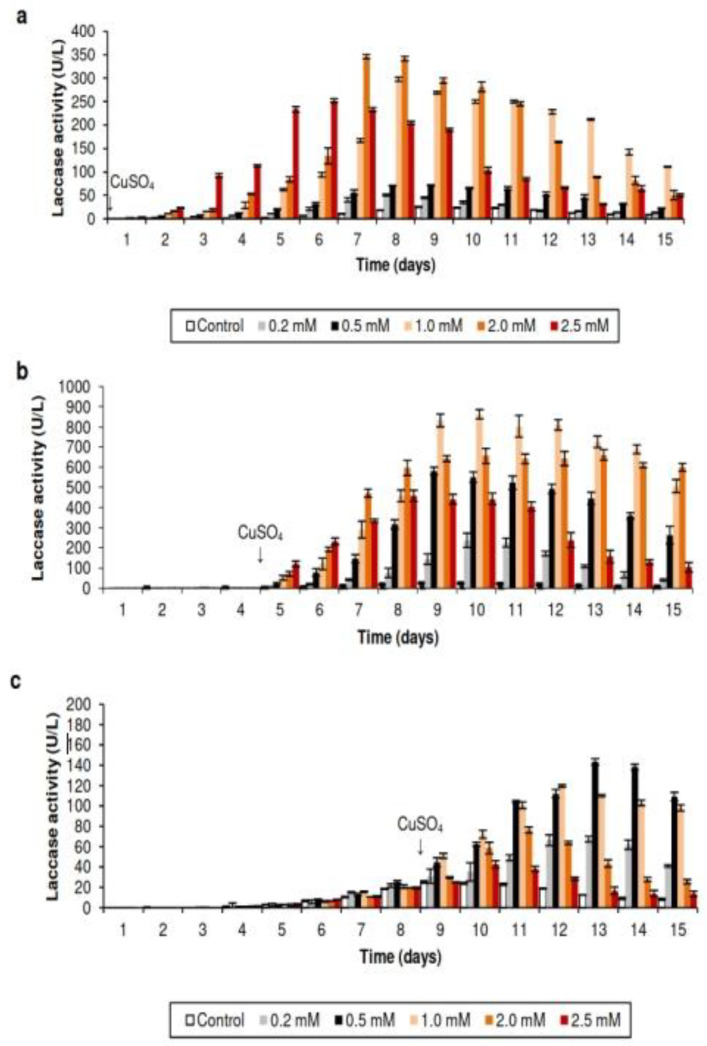
Effect of copper on the laccase production by *T. terrestris* Co3Bag1. Medium HCHN, supplemented with different concentrations of CuSO_4_ (0.2 to 2.5 mM), was added during three phases of incubation: (**a**) start of culture (0th day); (**b**) middle logarithmic phase (4th day); and (**c**) stationary phase (8th day).

The effect of copper on laccase production by *T. terrestris* Co3Bag1 grown in an HCHN medium was studied. Firstly, copper was added at the beginning of the culture (t = 0), the results of which are presented in Figure 3a. Maximum laccase activity (345.71 U/L) was observed with 2.0 mM CuSO_4_, which corresponds to a 13.7-fold increase than that observed for the culture without added copper (25.73 U/L), whereas maximum laccase activity of 251.98 U/L was recorded with 2.5 mM CuSO_4_; in both cases, enzymatic activity subsequently declined (after day 9 of culture). At 1.0 mM CuSO_4_, the maximum laccase activity was 297.53 U/L, and this enzymatic activity remained stable for the following 15 days of cultivation. The lowest laccase activities were obtained at 0.5 and 0.2 mM CuSO_4_, with enzymatic activities of 70.94 U/L and 50.29 U/L, respectively. Next, copper (from 0.2 to 2.5 mM CuSO_4_) was added to cultures on the 4th day of cultivation, corresponding to the middle logarithmic phase of growth (Figure 3b). The most significant laccase activity (862.76 U/L) was obtained with 1.0 mM CuSO_4_ on the 10th day of culture, which corresponds to a 33.5-fold increase compared to that observed without added copper (25.73 U/L), and a 32-fold increase compared to that registered with medium Zouari Mechichi et al. [21]. At 2.0 mM CuSO_4_, maximum laccase activity (661.19 U/L) was observed after the 13th day of culture and remained at this level for the next six days. In contrast, maximum laccase activity (458.0 U/L) was observed at 2.5 mM CuSO_4_ after the 8th day of culture and remained at this level for four days. At 0.2 mM CuSO_4_, maximum laccase activity (237.58 U/L, day 10) was notably lower than that observed in previous sets but 9.2-fold higher than the culture when copper was not added (25.73 U/L). Finally, the greatest production of laccase activity by *T. terrestris* Co3Bag1 was observed when copper (from 0.2 to 2.5 mM CuSO_4_) was added to cultures on the 8th day of cultivation, which corresponds to the stationary phase; the results obtained are shown in Figure 3c. Greater laccase activities were observed at both 0.50 mM and 1.0 mM CuSO_4_, with 143.37 U/L and 119.62 U/L, respectively, representing 5.5 and 4.6-fold more than that registered for the culture without added copper (25.73 U/L). The lowest laccase activities of 42.6 U/L, 76.63 U/L, and 67.42 U/L were obtained at 2.5, 2.0, and 0.2 mM CuSO_4_.

**Figure 4:**
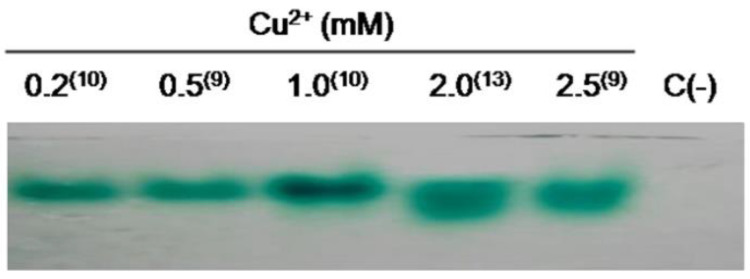
Zymogram analysis of laccase produced by *T. terrestris* Co3Bag1 in medium HCHN/1 mM CuSO_4_. Zymogram analysis of laccase in medium HCHN supplemented with CuSO_4_ (0.2 to 2.5 mM) in the middle logarithmic phase (4th day); samples obtained on the day of maximum laccase activity are shown in brackets. ABTS was used as the substrate.

Zymogram patterns of laccase activity induced by copper were determined using samples obtained from submerged fermentation of *T. terrestris* Co3Bag1 at the day of maximum laccase activity and supplemented with different concentrations of copper (0.2 to 2.5 mM CuSO_4_) during the middle logarithmic phase of incubation (4th day). The samples mentioned above were analyzed by native zymogram-PAGE using ABTS as the substrate (Figure 4). The zymogram laccase patterns showed a gradual increase in the signal of laccase activity for *T. terrestris* Co3Bag1 cultures when an amount between 0.2 to 1.0 mM of CuSO_4_ was added, exhibiting a greater signal at 1.0 mM CuSO_4_; in addition, at 2.0 and 2.5 mM CuSO_4_, more than one band was detected. Thus, the laccase zymogram patterns obtained are consistent with the previously described data obtained by the enzymatic assay, which suggests that high concentrations of copper (at 2.0 and 2.5 mM CuSO_4_) induced the expression of at least two types of laccase activity.

### 3.4. Growth and Laccase Production Kinetics of T. terrestris Co3Bag1 in Medium HCHN and CuSO_4_

**Figure 5:**
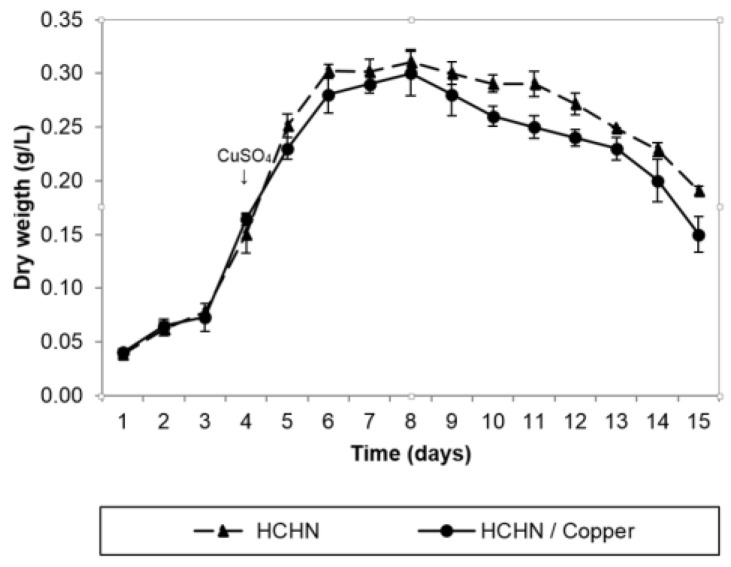
Growth kinetics of *T. terrestris* Co3Bag1 in HCHN/Copper. Growth kinetics in HCHN (▲, dotted line) and HCHN/Copper (1 mM CuSO_4_) (●, continuous line) medium. Growth was monitored by dry cell weight.

The growth of *T. terrestris* Co3Bag1 in a modified medium, designated as HCHN/1 mM CuSO_4_ (Figure 5), was compared to that registered for the culture with no added copper. It was found that both of these growth kinetics were typically logarithmic with a maximum biomass of 0.30 and 0.31 g/L, respectively, after the 8th day of incubation; then, a slight decrease in the growth of *T. terrestris* Co3Bag1 was observed at the end of cultivation in the presence of copper. These data suggest that adding 1 mM CuSO_4_ during the mid-logarithmic phase is not toxic to the fungus *T. terrestris* Co3Bag1. Thus, from the analysis of the results obtained in the study, concerning the effect of different carbon and nitrogen sources and the addition of copper during varying phases of the kinetics of growth, it was observed that the laccase activity increased from 26.9 U/L to 862.76 U/L, representing a 32-fold increase in enzymatic activity compared to that exhibited by *T. terrestris* Co3Bag1 grown in the original medium described by Zouari-Mechichi et al. [21].

### 3.5. Purification of TtLacA

**Figure 6:**
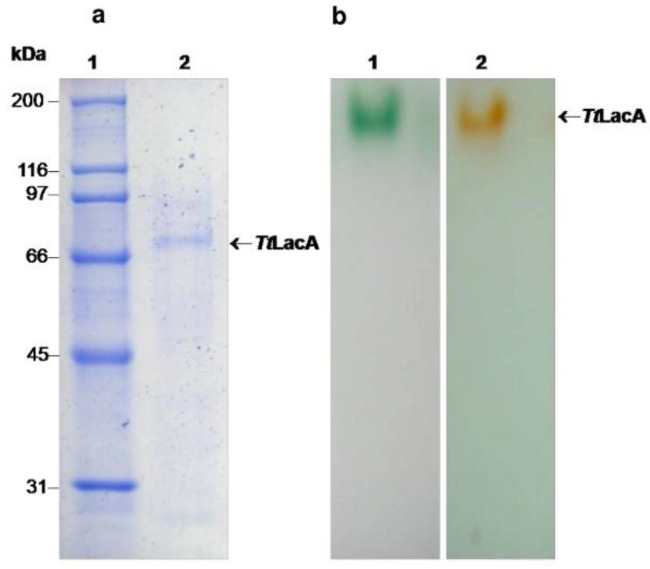
(**a**) Purification of *Tt*LacA of *T. terrestris* Co3Bag1. Lane 1, MW protein standards and lane 2, purified laccase. (**b**) Zymogram analysis of *Tt*LacA. Lane 1, ABTS and lane 2, 2,6-DMP, respectively, as the substrate.

In order to purify at least one of the molecules involved in the laccase activity produced by *T. terrestris* Co3Bag1, the cell-free culture supernatant of the fungus grown at 45 °C in the modified medium HCHN/1 mM CuSO_4_ was used as a source of laccase activity. All purification steps are summarized in Table 1. A laccase enzyme was purified 12.5-fold with a recovery yield of 32.5%. A single band with an estimated MW of 70 kDa was observed by SDS-PAGE analysis (Figure 6a) that exhibited laccase activity as shown by zymogram analysis using ABTS and 2,6-DMP as the substrates (Figure 6b). Thus, the 70 kDa protein was named *Tt*LacA.

### 3.6. Imobilization of TtLacA in Copper Alginate Beads

**Figure 7:**
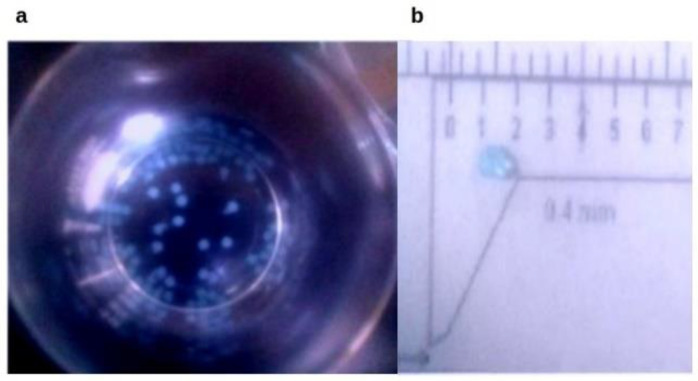
Copper alginate gel beads. (**a**) Photograph of the physical appearance and (**b**) size (measures approximately 1.0 mm in diameter) of immobilized *Tt*LacA in copper alginate gel beds synthesized.

Ca^2+^ is the most usually used metal ion for enzyme immobilization in alginate gel beads. At the beginning of this study, purified *Tt*LacA was immobilized in calcium alginate gel beads according to the method previously described [26]. However, although a 100% immobilization efficiency was observed for *Tt*LacA in calcium alginate gel beads, after five cycles of reuse the immobilized enzyme retained only 33% of the original activity (our unpublished results). For this reason, the purified laccase *Tt*LacA from *T. terrestris* Co3Bag1 was immobilized in copper alginate beads (diameter, 0.9 to 1.0 mm) (Figure 7), with an immobilization efficiency of 73.8%. Then, the *Tt*LacA immobilized in copper alginate gel beads and the free form of the enzyme was biochemically characterized.

#### 3.6.1. Optimal pH and pH Stability

**Figure 8:**
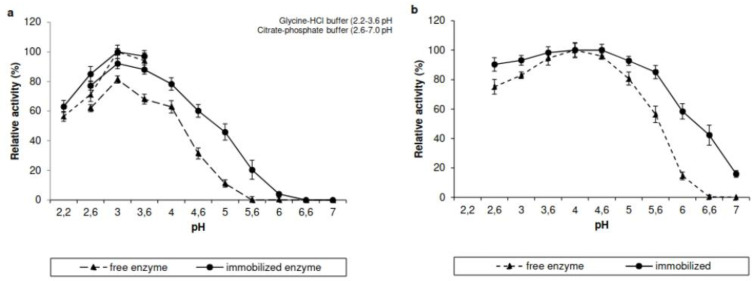
Optimal pH and pH stability of *Tt*LacA. Optimal pH and stability profile at different pH values for free (▲, dotted line) and immobilized (●, continuous line) *Tt*LacA in copper alginate gel beads. (**a**) The optimal pH was determined using two buffers (100 mM): glycine-HCl buffer (pH 2.2–3.6) and citrate-phosphate (pH 3.6–7.0). (**b**) the stability pH was determined using citrate-phosphate buffer (pH 2.6–7.0) at 25 °C for 50 min.

The effect of pH on the activities of free and immobilized laccase enzymes was determined in the pH range of 2.2 to 7.0 (Figure 8a). The optimum pH value of both free and immobilized *Tt*LacA was 3.0, and the corresponding activities for both preparations were higher in the glycine-HCl buffer. Immobilized *Tt*LacA exhibited some activity in the range of pH 5.6–6.0, whereas equivalent free *Tt*LacA activity was utterly lacking. The pH stability of free and immobilized *Tt*LacA was determined over a pH range from 2.6 to 7.0 (Figure 8b). Both free and immobilized forms of the enzyme retained at least 80% of relative activity in the pH range from 3 to 4.6 and maintained more than 50% of its initial activity in the pH range from 2.6 to 5.6. Immobilized *Tt*LacA maintained high enzyme activity (>50%) at pH 6.0 of its relative activity, whereas at pH 6.6 and 7.0, its relative activity decreased to 42.2 and 16%, respectively. The enzymatic activity of free *Tt*LacA decreased when the pH value reached 6.0.

#### 3.6.2. Optimal Temperature and Thermal Stability

**Figure 9:**
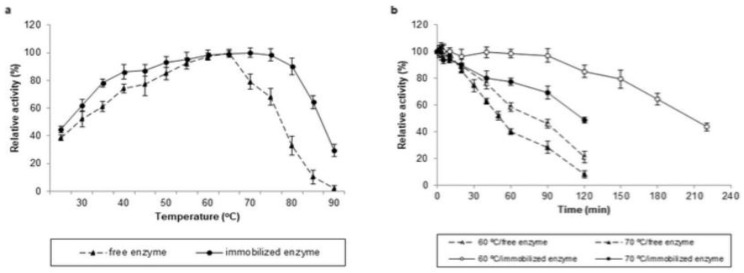
Optimal temperature and thermal stability of *Tt*LacA. Optimal temperature and stability profile at different temperatures for free (▲, dotted line) and immobilized (●, continuous line) *Tt*LacA in copper alginate beads. (**a**) The optimal temperature was determined at a range from 25–90 °C, at optimal pH (i.e., 3). (**b**) The thermal stability was determined at different temperatures in 100 mM glycine-HCl buffer (pH 3.0); open and filled symbols represent the temperature to 60 and 70 °C, respectively.

The effect of temperature on the activities of free and immobilized *Tt*LacA was determined at pH 3.0, over the temperature range of 25–90 °C (Figure 8a). The optimum temperatures were 65 and 70 °C for free and immobilized forms of *Tt*LacA, respectively; however, both of these displayed 70% of activity at temperature values from 40 to 70 °C and 50% of their maximum activity at the range of 35 to 75 °C. Likewise, the thermal stability of free and immobilized *Tt*LacA was evaluated at temperature values of 60 and 70 °C at optimal pH (Figure 9b). Immobilized *Tt*LacA manifested 2.3-fold greater thermal stability at 60 (t_1/2_ of 191.5 min) and 70 °C (t_1/2_ of 117.2 min) than that displayed by the free form of the enzyme at the same temperature values, with half-lives (t_1/2_) of 82.9 min and 50.3 min at 60 and 70 °C, respectively (Figure 9b).

#### 3.6.3. Kinetic Parameters

The calculated *K*_m_ values of free and immobilized *Tt*LacA were 260 and 450 µM, respectively, indicating that the free enzyme had a 1.7-fold greater affinity for the substrate than the immobilized enzyme. The *k*_cat_ of the free and immobilized *Tt*LacA were 13.73 and 6.35 s^−1^, respectively, indicating that the free enzyme had a 2.16-fold greater catalytic efficiency than immobilized *Tt*LacA.

#### 3.6.4. Effects of Metal Ions, EDTA, and NaN_3_ on *Tt*LacA Activity

The effect of metal ions and the agents EDTA and NaN_3_ (1 and 10 mM each) on the activity of free and immobilized *Tt*LacA were evaluated under optimal assay conditions, and the corresponding results obtained are summarized in Table 2. The laccase activity of the free *Tt*LacA decreased from 10 to 90% with a final concentration of 1 mM of all tested metal ions, except for metal ion Cu^2+^ (1 mM), where an increase of 28% was recorded. In contrast, the laccase activity of the free *Tt*LacA decreased over a range from 35 to 84% in the presence of 10 mM for each of all the metal ions tested, except for 10 mM Cu^2+^, where a slight increase was observed. The free *Tt*LacA was entirely inactivated by Hg^2+^ (1 and 10 mM) and also Fe^2+^ (10 mM). Immobilized *Tt*LacA exhibited greater tolerance in the presence of metal ions. The laccase activity of the immobilized *Tt*LacA decreased from 6 to 86% with a final concentration of 1 mM for all metal ions tested, except for the Cu^2+^ (1 mM), for which the activity of immobilized *Tt*LacA was not affected; in contrast, there was a decrease of 6% in the presence of the Cu^2+^ (10 mM). The laccase activity of the immobilized *Tt*LacA decreased by 6 to 90% in the presence of 10 mM for all metal ions tested, except for Hg^2+^ (10 mM), where immobilized *Tt*LacA was entirely inactivated.

The laccase activity of free *Tt*LacA (32 and 100%) and immobilized (21 and 70%) forms of the enzyme was inhibited by EDTA to final concentrations of 1 and 10 mM, respectively. Finally, free (96 and 100%) and immobilized (86 and 94%) *Tt*LacA activity was strongly inhibited by NaN_3_ at 1 and 10 mM, respectively.

### 3.7. Storage Stability of the Free and Immobilized TtLacA

**Figure 10:**
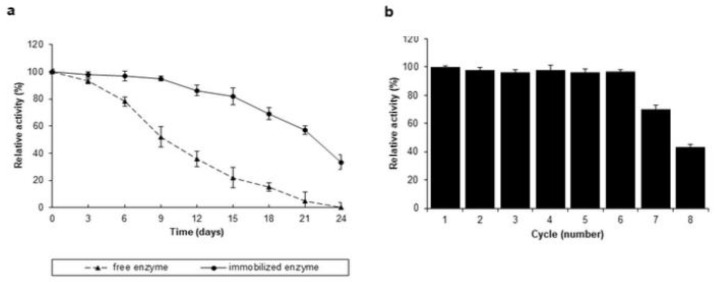
(**a**) Storage stabilities of *Tt*LacA in copper alginate beads. Storage stabilities at 4 °C in glycine-HCl buffer (pH 3.0) values for free (▲, dotted line) and immobilized (●, continuous line) *Tt*LacA in copper alginate gel beads. The laccase activity was assayed under optimal conditions. (**b**) Reusability of immobilized *Tt*LacA in copper alginate gel beads. The reusability of the immobilized enzyme was determined by assaying the laccase activity in each cycle after incubation at 60 °C, using ten copper alginate gel beads per cycle.

Storage stability represents one of the most useful characteristics of immobilized enzymes for industrial applications. The *Tt*LacA immobilized in copper alginate gel beads, or the free form of the enzyme, were stored in the glycine-HCl buffer (100 mM, pH 3.0) at 4 °C for 24 days. As shown in Figure 10a, the activity of free *Tt*LacA started to decrease after the first three days. In contrast, the activity of immobilized laccase did not exhibit any considerable decline in activity for up to 15 days. The free enzyme retained 35.64% of its initial activity by the 12th storage day and lost the activity entirely by the 24th storage day. In contrast, the activity of immobilized laccase was 86.1% and 33.4% of its initial activity over the same period.

### 3.8. Reusability of Immobilized TtLacA

The reusability profile of immobilized *Tt*LacA in copper alginate gel beads is shown in Figure 10b. The results revealed up to 95% continued activity for six consecutive cycles. Following cycle number seven, the activity of immobilized *Tt*LacA was reduced to 70.3%, which then decreased further to 43.6% at the end of the 8th consecutive cycle of reuse.

### 3.9. TtLacA Partial Sequencing and Sequence Analysis of the Multicopper Protein from T. terrestris NRRL 8126

**Figure 11:**
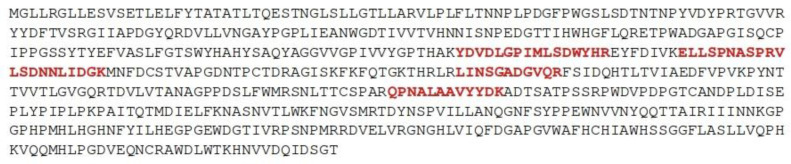
The amino acid sequence of laccase from *T. terrestris* NRRL 8126 (UniProtKB: G2R0D5). Five peptides from *Tt*LacA are highlighted in red boldface.

The amino acid sequence of five *Tt*LacA peptides showed 100% similarity to an equivalent sequence reported within a multicopper protein from *T. terrestris* NRRL 8126 (UniProtKB: G2R0D5) (Figure 11). In addition, the peptide QPNALAAVYYDK displayed 91.7% similarity to a laccase from *Rhynchosporium commune* (UniProtKB: A0A1E1KUV9); whereas the peptide YDVDLGPIMLSDWYHR exhibited 93.3% similarity to laccase from *Trichoderma* sp. (UniProtKB: C5H3G0). The peptides VLSDNNLIDGK and LINSGADGVQR showed 90.9% similarity to laccase with a copper-binding domain from *Myceliophthora thermophila* (UniProtKB: G2Q560).

**Figure 12:**
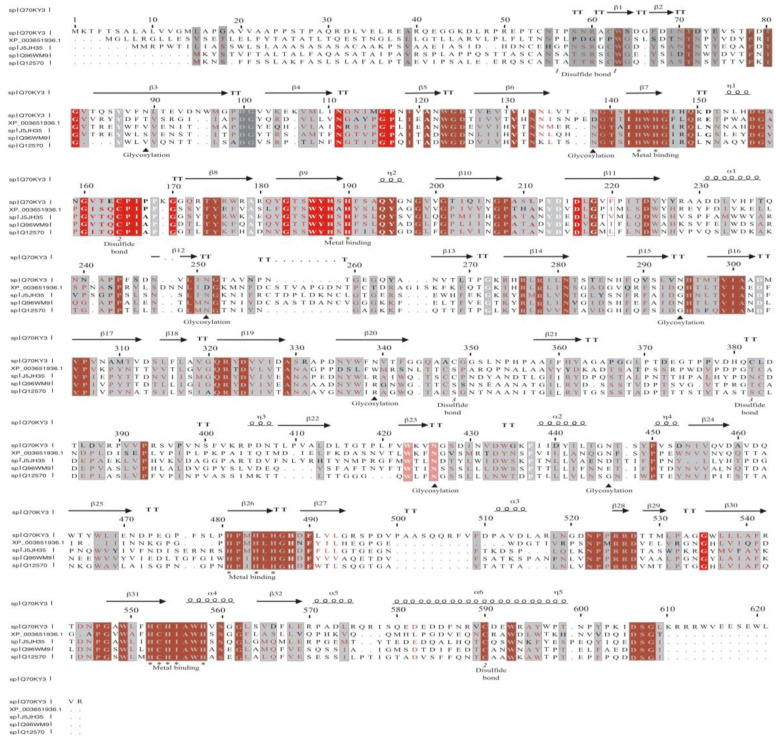
Multiple sequence alignment and secondary structure element assignment. The alignment included oxidoreductase from *Beauveria bassiana* ARSEF 2860, laccases from *Botrytis cinerea* (UniProtKB: Q12570, LAC1_BOTFU; UniProtKB: Q96WM9, LAC2_BOTFU), the multicopper protein from *T. terrestris* NRRL 8126 (THITHE NRRL 8126; UniProtKB: G2R0D5), and 3D crystal structure laccase from *M. albomyces* MaLac1 (UniProtKB: Q70KY3, PDB code 3QPK). The metal-binding sites were manually determined. The helices are marked as alpha or beta based on the automatic assignment according to the template of the PDB code 3QPK protein structure in the program ESPript [28].

Figure 12, shows the alignment of the multicopper-like protein (XP_003651936.1) from *T. terrestris* NRRL 8126 (UniProtKB: G2R0D5) with the sequences from BLAST analysis. In addition, six α-helices, 32 β-sheets, and four metal-binding sites (HWHG, HSHF, HPMHLHGHDF, and HCHIAWH) were identified as multicopper from *T. terrestris* NRRLL 8126 (UniProtKB: G2R0D5) with a laccase from the ascomycete fungus *M. albomyces* MaLac1 *(*UniProtKB: Q70KY3), which had been purified and crystallized with an intact trinuclear copper site (PDB code 3QPK).

**Figure 13:**
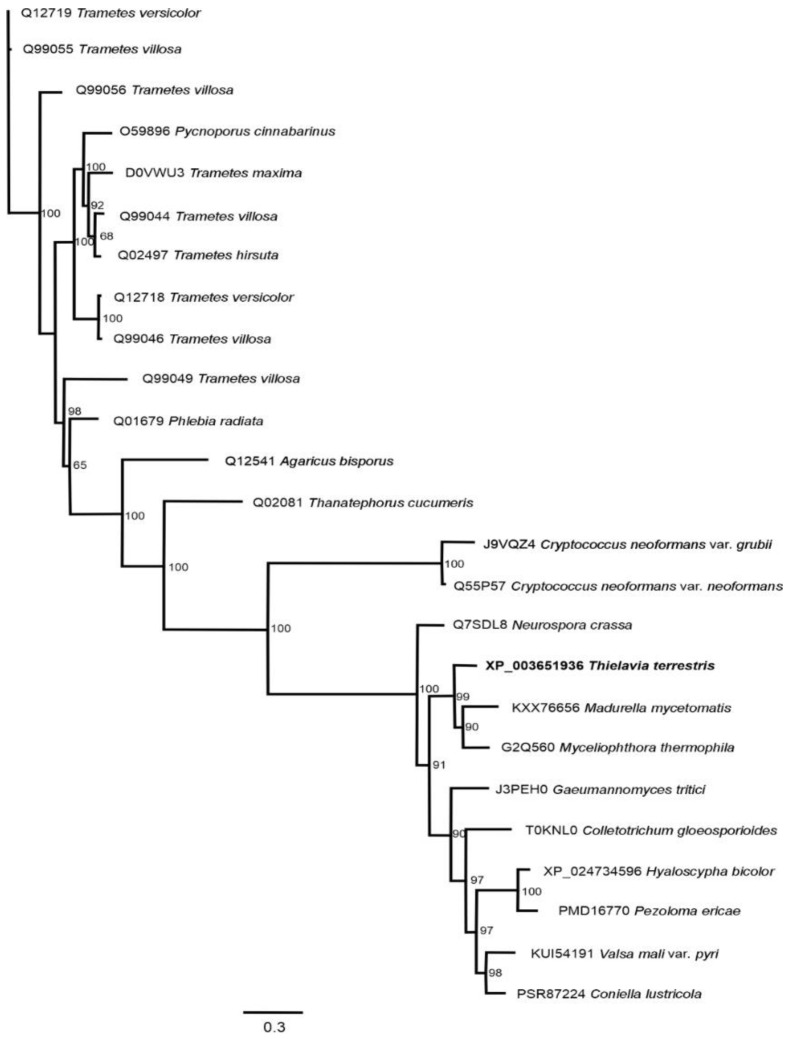
Maximum-likelihood tree (**1**) of multicopper oxidase from *T. terrestris* NRRL 8126 (UniProtKB: G2R0D5) with other multicopper oxidases and laccases. The analysis involved 25 amino acid sequences, accession numbers of UniProtKB, and the NCBI database; these are shown in the tree branches. *T. versicolor* (UniProtKB: Q12719) was used as an outgroup.

The sequence of putative multicopper oxidase from *T. terrestris* NRRL 8126 (UniProtKB: G2R0D5) was grouped with multicopper oxidase or laccases from ascomycetes and basidiomycetes with the greatest log-likelihood (-16.104.02) and with a strong nodal support value (100); the laccase from *T. terrestris* NRRL 8126 (UniProtKB: G2R0D5) was grouped with laccases from *Madurella mycetomatis* (KXX76656) and *M. thermophilA* (UniProtKB: G2Q560) as shown in Figure 13. Taking a second phylogenetic approach, the laccase from *T. terrestris* NRRL 8126 (UniProtKB: G2R0D5) showed a relationship with a laccase from *T. terrestris* (A0A3S4AJJ5). A relationship with plant laccases from the Magnoliophyta phylum was observed (66%), particularly *Arabidopsis thaliana* and *Oriza sativa* laccases (Supplementary Figure S1).

## 4. Discussion

Laccases are known to be efficient, sustainable, and environment-friendly biocatalysts, which can be considered appropriate for industrial applications of these enzymes [33]. Unfortunately, laccase yields are low for most laccases producing species; a deficiency which can be solved by selecting new organisms with laccase synthesis ability or optimizing culture conditions [33]. In this study, we focused on the effect of certain factors that increase a novel laccase activity by the thermophilic biomass-degrading ascomycete *T. terrestris* Co3Bag1. Our preliminary culture of *T. terrestris* Co3Bag1, grown on the liquid medium previously described [21], was able to produce laccase activity, but with it a low oxidation level on ABTS; hence, the culture conditions were modified, and their effect in the production of laccase activity by *T. terrestris* Co3Bag1 was evaluated.

Firstly, we assessed the production of laccase activity by *T. terrestris* Co3Bag1 with different carbon and nitrogen sources. Fructose was the best carbon source with a 1.5-fold increase in the laccase activity produced by *T. terrestris* Co3Bag1, even though glucose has been considered the best carbon source for laccase production [34]. Our findings agree with an increase in laccase activity produced by the basidiomycete CECT 20197 with fructose as the carbon source, compared to that obtained with glucose [35]. Likewise, fructose supplementation also showed an increase in the laccase activity produced by *Trametes hirsuta* MTCC 11397 [4] and *Agaricus* sp. [36]. Similarly, using of inorganic sodium nitrate as a sole nitrogen source, there was a 3.18-fold enhancement in the laccase activity produced by *T. terrestris* Co3Bag1. Our findings agree that an increase in the laccase activity produced by *Pleurotus eryngii* was obtained after adding sodium nitrate as the only nitrogen source under solid-state bioprocessing [37]. In contrast, it has been reported that laccase production by *Myrothecium roridum* in the presence of yeast extract was similar to that achieved in a medium containing sodium nitrate [38].

Although it is difficult to identify the best carbon and nitrogen sources for a particular fungus, it is generally accepted that the production of laccase by fungi not only depends on the variable influence of carbon and nitrogen sources but also on their relative concentrations [39]. The production of laccase activity by *T. terrestris* Co3Bag1 was evident in all tested culture media; however, the greatest laccase activity was displayed when fructose and sodium nitrate were maintained at a high concentration (HCHN). Concurring with our data, an improvement in laccase production with high carbon-nitrogen concentrations in the fermentation medium of *T. hirsuta* MTCC 11397 was described [4]. In contrast, Schneider et al. [40], reported that low carbon-nitrogen concentrations caused greater laccase production by *Marasmiellus palmivorus* VE111. In contrast, elevated laccase activity has been observed using high carbon and low nitrogen concentrations in *Coprinus comatus* [41].

Because laccase activity production can be enhanced among a great variety of ligninolytic fungi by the addition of certain alcohols, aromatic compounds, metal ions, and, in particular, Cu^2+^ [6,8], the effect of copper in the production of laccase by *T. terrestris* Co3Bag1 was evaluated in this work. Notably, the addition of copper (1 mM) in the middle logarithmic phase of growth of this fungus had a remarkable stimulatory effect, corresponding to a 32-fold increase in the production of laccase activity by *T. terrestris* Co3Bag1; likewise, zymogram analysis revealed a single band of activity in the presence of 1 mM CuSO_4_, whereas two bands of activity seem to be induced with 2.0 and 2.5 mM CuSO_4_.

An increase of 10-fold and 1.4-fold in laccase activity has been registered at 1 mM CuSO_4_ in *Pleurotus ostreatus* [42] and white rot fungus WR-1 [43], respectively. Variations in terms of the effect of copper addition on laccase activity and isoenzymatic patterns from *Pleurotus sajor-caju*, as laccase activity increased 2.3 and 1.3-fold in cultures supplemented with 0.5 and 1 mM CuSO_4_, respectively, and correlation with two isoenzymes of 65 kDa and 35 kDa shown by zymogram analysis has been reported [44]. In contrast, greater laccase production has been observed with low copper concentrations, e.g., the production of laccase activity by *Agaricus blazei* U2-4 in the culture containing 150 µM CuSO_4_ was 0.96-fold higher than that in the control medium, lacking copper [45].

Inhibition of mycelial growth associated with fungal laccase activity and the addition of copper to the medium culture has been previously reported by Zhu et al. [6], who observed the greatest laccase activity after adding copper, but only during the middle logarithmic fermentation phase (5th day); however, mycelial growth was inhibited by approximately 27%. It has been suggested that reduced fungal growth might be the result of oxidative stress in high copper concentration and might be responsible for late transcriptional induction [46]. In this work, the growth kinetics of *T. terrestris* Co3Bag1 (day 8, maximal activity) was slightly lower (6.7%) in the presence of 1 mM CuSO_4_, when added during the middle logarithmic phase of growth (day 4) than that reached in a free copper culture; thus suggesting a slightly toxic effect of 1 mM CuSO_4_ on the mycelial growth of this fungus.

*Tt*LacA of 70 kDa was purified from *T. terrestris* Co3Bag1, grown at 45 °C on a modified medium known as HCHN/1 mM CuSO_4_; hence, *Tt*LacA represents one of the molecules involved of the laccase activity produced by this fungus under the above-mentioned conditions. Fungal laccases with molecular mass ranging from 48 to 84 kDa [47] (see Table S1) have been reported, such as laccases from *Thielavia* sp. (70 kDa) [48], *Trametes polyzona* WRF03 (66 kDa) [49], *Trametes* pubescens (68 kDa) [50], *Paraconiothyrium variabile* (84 kDa) [51], and *Ganoderma australe* (48 kDa) [52]. Purified *Tt*LacA was immobilized by the entrapment method in copper alginate gel beads; then, the free and immobilized *Tt*LacA were biochemically characterized.

Entrapment is a method of immobilization where the enzyme is retained in a porous solid matrix [16]. The use of alginate has proved a good strategy for the enzyme entrapment method; the alginate can be prepared as hydrogels shaped like beads in the presence of divalent cations such as calcium [53]; although, it is apparent that copper has a more significant affinity to alginate than that to calcium during alginate gelation [54]. In this work, *Tt*LacA was immobilized in copper alginate gel beads with 73.8% efficiency. Similarly, an immobilization efficiency of 75, 80 and 85% have been reported for laccases from *Cyberlindnera fabianii* [10], *Bacillus* sp. [26], and *T. versicolor* [55], respectively, in copper alginate gels beads.

Free and immobilized *Tt*LacA displayed optimal activity at pH 3.0, although the enzymes were active over the pH range from 2.2 to 4.0. According to Baldrian et al. [56], the fungal laccases display the highest catalytic activity in the acidic pH range; although change depended on the kind of substrate, e.g., pH values lower than 4.0 are typical for ABTS oxidation. Concurring with our data, free laccases from *Chaetomium* sp. [57] and *Xylaria* sp. [58] exhibited an optimum pH of 3.0. In contrast, optimal pH for activity of 2.2 and 6.0 have been observed for free laccases from Gymnopus luxurians [59] and Trichoderma harzianum HZN10 [60], respectively. An optimal pH value of 3.0 for both free and immobilized laccase in carbon nanotube membranes from *M. thermophila*, has been reported [61]. In addition, the same optimal pH value (pH 6.0) for both free and immobilized laccase in calcium alginate gel beads from *T. harzianum* HZN10 was observed [60] (see Table S1). The laccase activity of free and immobilized *Tt*LacA at pH values above 4.0 decreased gradually. Similar results have been reported for immobilized laccase in calcium and copper alginate (Ca-AIL and Cu-AIL) with a decline in enzymatic activity at pH values above 5.0 [10]. It has been proposed that when a laccase is in a neutral or alkaline environment, the decrease in laccase activity is due to the binding of a hydroxide anion to the T2/T3 coppers of laccase, which disrupts the internal electron transfer between T1 and T2/T3, thus inhibiting enzyme activity [62]. Assays with pH stability indicated that free and immobilized *Tt*LacA remained active under acidic conditions in the pH range from 2.6 to 5.0. Similarly, a laccase from *Myrothecium verrucaria* NF-05 was stable in the pH range of 3.0–7.0 [63]. Contrastingly, free and immobilized laccase from *M. thermophila* was stable at alkaline pH [61].

Immobilized *Tt*LacA displayed optimal activity at 70 °C, which corresponds to an upward shift of 5 °C compared to the free form of *Tt*LacA, with optimal activity at 65 °C. According to Vieille and Zeikus [64], *Tt*LacA may be considered a thermophilic enzyme. The optimal temperature of free *Tt*LacA (65 °C) exceeded that reported for the laccases from *Cerrenea unicolor* (45 °C) [65], *P. variabile,* and *Shiraia* sp. (50 °C) [51,66], but were similar to those reported for laccases from *Thielavia* sp. [48], *Chaetomium* sp. [57], and *Trametes troggi* (60 °C) [67], and less than the laccase from *Marasmius quercophilus* (80 °C) [68]. Moreover, similar results of a temperature shift between free and immobilized enzymes have been reported for immobilized laccases in copper alginate gel beads (40 to 50 °C) and calcium alginate gel beads (40 to 60 °C) from *C. fabianii* [10] (see Table S1). The thermal stability of the free form of *Tt*LacA at 70 °C was more significant than those previously reported for laccases from *Shiraia* sp. [66] and a laccase from *Chaetomium* sp. [57]; in contrast, at 60 °C, *Tt*LacA displayed less stability than that reported for laccases from *Shiraia* sp. [66] and from *Thielavia* sp. [48]. Notably, the laccase *Tt*LacA immobilized in copper alginate gel beads showed more thermal stability than the free form of the enzyme; thus suggesting that the copper alginate matrix preserved the conformational stability and catalytic activity of the laccase *Tt*LacA. Notably, thermal stabilities at temperatures above 70 °C were not assessed because, as reported previously, the pearls melt at elevated temperatures [26] (see Table S1).

The kinetic parameters for purified *Tt*LacA were determined using ABTS as the substrate (see Table S1). The *K*_m_ value of *Tt*LacA is similar to the *K*_m_ value reported for the laccase from ascomycete *P. variabile* [51]; this was higher than those reported for the laccases from *C. fabianii* [10] (see Table S1). The *V*_max_ value of *Tt*LacA was higher than the *V*_max_ value from *P. variabile* [51] (see Table S1). The *K*_m_ value of immobilized *Tt*LacA was 1.7-fold greater than free laccase; thus indicating lower substrate affinity than that displayed by the free form of the enzyme. In contrast, the *V*_max_ value of immobilized *Tt*LacA was 1.2-fold lower than that of the free laccase. Bagewadi et al. [60] observed an increase in the *K*_m_ value upon immobilization in copper alginate gel beads for laccase from *T. harzianum* HZN10; the *V*_max_ value of immobilized laccase was higher than that of free laccase. Other research by Mohammadi et al. [69] reported similar *K*_m_ values for free and immobilized laccase on epoxy-functionalized silica from *M. thermophila*. An increase in the *K*_m_ value and a decrease in the *V*_max_ value upon immobilization of *Tt*LacA in copper alginate gel beads may be attributed to a change in the steric hindrance of an enzyme active site, possibly due to diffusion limitation of the substrate into the gel matrix and decreased protein flexibility displayed by entrapped enzymes, which may result in a decrease in catalytic efficiency, as has been described [60,70] (see Table S1).

The metal ions play a functional role for metalloenzymes implicated in the catalytic process, including electron transfer and substrate recognition/binding [71]; therefore, metal ions can support the conformation of its active site or well acting as a competitive inhibitor of electron donors by blocking the access of substrates to the T1 site [72]. It has been reported that copper ions are involved in the catalytic mechanism of typical laccases [2]. Free *Tt*LacA activity was only affected positively by the metal ion Cu^2+^. Interestingly, an increase of 28% was observed for the laccase activity of free *Tt*LacA in the presence of 1 mM Cu^2+^. In contrast, a decrease of 6% was registered when the immobilized *Tt*LacA was challenged with 10 mM Cu^2+^; thus suggesting that immobilization on copper alginate gel beds does not eliminate the inhibitory effect of the metal ion Cu^2+^ on laccase activity of *Tt*LacA (see Table S1). This behavior is similar to that described for a fungal laccase from *Peniophora* sp. [73], which shows an increase in the relative activity in the presence of Cu^2+^. Similarly, an increase in the enzymatic activity of laccases ThLacc-S from *T. hirsuta* [74] and Tplac from *Trametes pubescens* [72] was enhanced with the ion Cu^2+^ at 25 mM. Free *Tt*LacA was strongly inhibited by Fe^2+^ and Hg^2+^. This finding is similar to other studies, which reported that Fe^2+^ inhibits laccase activity [48,57,58]. This contrasts with the stimulatory effect of Fe^2+^ for the laccases from *Methylobacterium extorquens* [75], *T. hirsuta* MX2 [76], *G. austral* [52], and T. polyzona WRF03 [49]. Strong inhibition of laccases from *T. hirsuta* [74] and, *T. harzianum* S7113 [77] by Hg^2+^ has been registered. Inhibition by Hg^2+^ ions might be due to the strong binding affinity of these ions for sulfhydryl groups, present on histidine residues of the catalytic site involved in binding the four copper atoms, thus causing enzyme deactivation [2,78]. *Tt*LacA was affected by the presence of EDTA, principally at the high concentration tested, which indicated the importance of divalent cations for the enzyme activity to be a metalloenzyme [71]. Other results exhibited better relative activity in the presence of EDTA [59,76]. Interestingly, Lorenzo et al. [79], observed a significant inhibitory effect by EDTA on laccase when DMP or syringaldazine were used as a substrate; thus, suggesting the type of substrate involved in the inhibitory effect by EDTA on laccase [72,79]. Similar inhibition of free *Tt*LacA activity by NaN_3_ (1 mM) was reported for laccase from *Chaetomium* sp. [57] and *Thielavia* sp. [48]; this inhibition is probably caused by the binding of NaN_3_ to T2 and T3 copper sites, which blocks the electron transfer from the T1 copper site and reduces the molecular oxygen of laccase, thereby reducing catalytic activity [80].

The storage stability and reusability of immobilized laccase in copper alginate gel beads were also studied because of their importance in industrial applications in terms of reducing processing costs. *Tt*LacA retained 50% of its initial activity after 9 days of storage at 4 °C. In contrast, the stability of immobilized laccase in copper alginate gel beads was enhanced, and it retained more than 50% for 21 days under the same storage conditions. This observation is similar to that of Sondhi et al. [26], who reported that immobilized laccase from *Bacillus* sp. in copper alginate gel beads retains more than 95% initial activity after 15 days at 4 °C (see Table S1). The reusability of the immobilized enzyme is a necessary factor for assessing the value of immobilization. Immobilized *Tt*LacA retains 95% of its initial activity after the 6th cycle of reuse to 60 °C. This result was higher than that previously reported for immobilized laccase in copper alginate gel beads, which retains 100% of its activity after the 4th cycle repetition [26]. In agreement with our data, a similar pattern of rising storage stability after immobilization has been reported by [81] and [50] (see Table S1). Although enzyme immobilization in copper alginate gel beads has been successfully employed for the immobilization of laccase TtLacA (this work) and immobilization of other bacterial [26] and fungal [60] laccases, the potential consequences of using Cu^2+^ as the gelling agent, rather than another divalent cation such Ca^2+^, must be evaluated per each target enzyme, because (i) the metal ion Cu^2+^ may inhibit the activity of some immobilized enzymes [79] and (ii) the metal ion Cu^2+^ shows greater affinity to alginate than Ca^2+^ [54]; therefore, it could give rise to a competition between the binding of copper to the alginate rather than to a metalloprotein, such as a multicopper oxidase.

Based strictly on the amino acid sequence of the five peptides generated from purified *Tt*LacA and compared to some other reported fungal laccases, *Tt*LacA exhibited 100% similarity to the multicopper protein from *T. terrestris* NRRL8126 (UniProtKB: G2R0D5), which was later compared to a crystallized laccase from the ascomycete *M. albomyces* MaLac1 [82]. This analysis showed four conserved copper-binding motifs, Cu I (HWHGFFQ), Cu II (HSHLSTQ), Cu III (HPFHLHGH), and Cu IV (HCHIDWHL), typical in fungal laccases [83]; thus supporting the suggestion that the multicopper protein is a typical fungal laccase. This suggestion is further corroborated by a close phylogenetic relationship between the multicopper protein from *T. terrestris* NRRL 8126 (UniProtKB:G2R0D5) and laccase from *M. mycetomatis* (KXX76656). According to the phylogenetic analyses reported by Sande [84], *M. mycetomatis* (KXX76656) belongs to the order Sordoriales, and this fungus is most closely related to *Chaetomium thermophilum*. Moreover, *T. terrestris* NRRL 8126 (UniProtKB: G2R0D5) manifests a closer phylogenetic relationship with laccase from *T. terrestris* (A0A3S4AJJ5); thus, phylogenetic analysis confirms that laccases from ascomycetes and basidiomycetes are independent clades. Evidently, these fungal laccases are separated from the laccases of plants, which is consistent with the taxonomical classification [85]. Interestingly, Janusz et al. [1] reported a greater similarity between laccases produced by ascomycetes, plants, bacteria, and insects than between fungal enzymes. According to Lombard et al. [86], laccases are classified within the AA1 enzyme family of auxiliary activities using the CAZY classification system. Then, an expansion of the enzymatic repertoire of the CAZy database to integrate auxiliary redox enzymes was reported by Levasseur et al. 2013 [87]. In this study, laccases and ferroxidases from basidiomycetes comprise the AA1 subfamilies 1 and 2, respectively, whereas laccases from ascomycota, with multicopper like oxidases, have been categorized into AA1 subfamilies 3 [87]. This suggests that laccase *Tt*LacA from the thermophilic ascomycete fungus *T. terrestris* Co3Bag1 may be categorized as a member of the AA1-3 subfamily.

## 5. Conclusions

This report describes a 32-fold improvement in laccase activity produced by the ascomycete thermophilic biomass-degrading fungus *T. terrestris* Co3Bag1 in response to high-concentration fructose and sodium nitrate as carbon-nitrogen sources and the addition of copper ions in the middle logarithmic phase of growth. A 70 kDa novel thermophilic laccase (named *Tt*LacA) was purified from the cell-free culture supernatant from *T. terrestris* Co3Bag1, and grown under improved conditions for the production of laccase activity. *Tt*LacA was identified by a bioinformatic analysis of a partial amino acid sequence as multicopper oxidase and classified as a member of the asco-laccase AA1-3 subfamily. Hence, *Tt*LacA represents one of the molecules involved in the laccase activity produced by this fungus under improved conditions for laccase production. Purified *Tt*LacA was successfully immobilized in copper alginate gel beads, exhibiting (i) a widened pH-activity profile, (ii) an optimum temperature that was shifted from 65 to 70 °C, (iii) an improved stability to pH and temperature, and (iv) good tolerance of metal ions and other inhibitory agents, compared to those observed for the free form of the enzyme. Additionally, immobilized *Tt*LacA retained 95% initial activity for six consecutive reuse cycles at 60 °C. In addition, 86% of the initial activity is retained after 12 days of storage at 4 °C. Based on the biochemical properties manifested in immobilized *Tt*LacA from *T. terrestris* Co3Bag1, it may be an efficient enzyme for dye decolorization and other industrial applications carried out at high temperatures or under acidic conditions. Furthermore, this represents the first report about the immobilization of a thermophilic laccase from a member of the genus *Thielavia*.

## Figures and Tables

**Table 1:** Summary of purification of *Tt*LacA.

Purification Step	Total Activity (U)	Total Protein (mg)	Specific Activity (U/mg)	Purification Fold	Yield (%)
Crude extract	1.195	8.074	0.148	1.0	100
Ultrafiltration (50 kDa)	0.857	0.692	1.238	8.4	71.7
AEC *	0.389	0.210	1.852	12.5	32.5

* Anionic exchange chromatography.

**Table 2:** Effect of metal ions and inhibitors on the activity of free and immobilized *Tt*LacA.

	Relative Activity (%)			
	Free Laccase		Immobilized Laccase **	
Concentration	1 mM	10 mM	1 mM	10 mM
**Metal ions**				
Control *	100	100	100	100
Na^+^	89.32 ± 1.3	63.08 ± 2.2	93.12 ± 1.8	77.10 ± 1.6
Cu^2+^	128.21 ± 2.3	105.70 ± 5.9	101.2 ± 2.5	94.23 ± 3.1
Ca^2+^	90.00 ± 0.6	65.11 ± 3.4	93.90 ± 0.9	81.21 ± 1.2
Co^2+^	54.65 ± 3.6	16.27 ± 2.3	70.85 ± 1.4	45.46 ± 0.9
Zn^2+^	74.21 ± 1.4	55.18 ± 6.1	86.49 ± 1.5	75.18 ± 0.8
Mg^2+^	88.37 ± 1.7	60.04 ± 2.4	90.03 ± 4.1	83.72 ± 2.2
Fe^2+^	10.42 ± 4.3	0	50.06 ± 3.2	10.30 ± 1.8
Hg^2+^	0	0	14.30 ± 3.5	0
**Inhibitors**				
EDTA	67.51 ± 2.8	0	79.37 ± 2.2	30.16 ± 3.1
NaN3	3.80 ± 0.3	0	14.31 ± 2.3	5.90 ± 1.9

* Activity of purified *Tt*LacA with no additions; data are mean ± SD for three measurements (n = 3). ** Immobilized laccase in copper alginate beads.

## Data Availability

Not applicable.

## Supplementary Materials

The following supporting information can be downloaded at: https://www.mdpi.com/article/10.3390/jof9030308/s1, Table S1. Comparison biochemical properties of free and immobilized *Tt*LacA and others laccases; Figure S1. Maximum-likelihood tree (2) of multicopper oxidase from *T. terrestris* NRRL 8126 (UniProtKB: G2R0D5) with other multicopper oxidases and laccases.
