# Supplementary material for: Improvement of Laccase Production by Thielavia terrestris Co3Bag1. Enhancing the Bio-Catalytic Performance of the Native Thermophilic TtLacA via Immobilization in Copper Alginate Gel Beads
Source: J Fungi (Basel). 2023 Feb 28;9(3):308. doi: 10.3390/jof9030308 (PMC10057929; doi:10.3390/jof9030308)
Supplement: Supplementary file 1 [file jof-09-00308-s001.zip › 3.Figure S1_jof-2142823 .pdf]

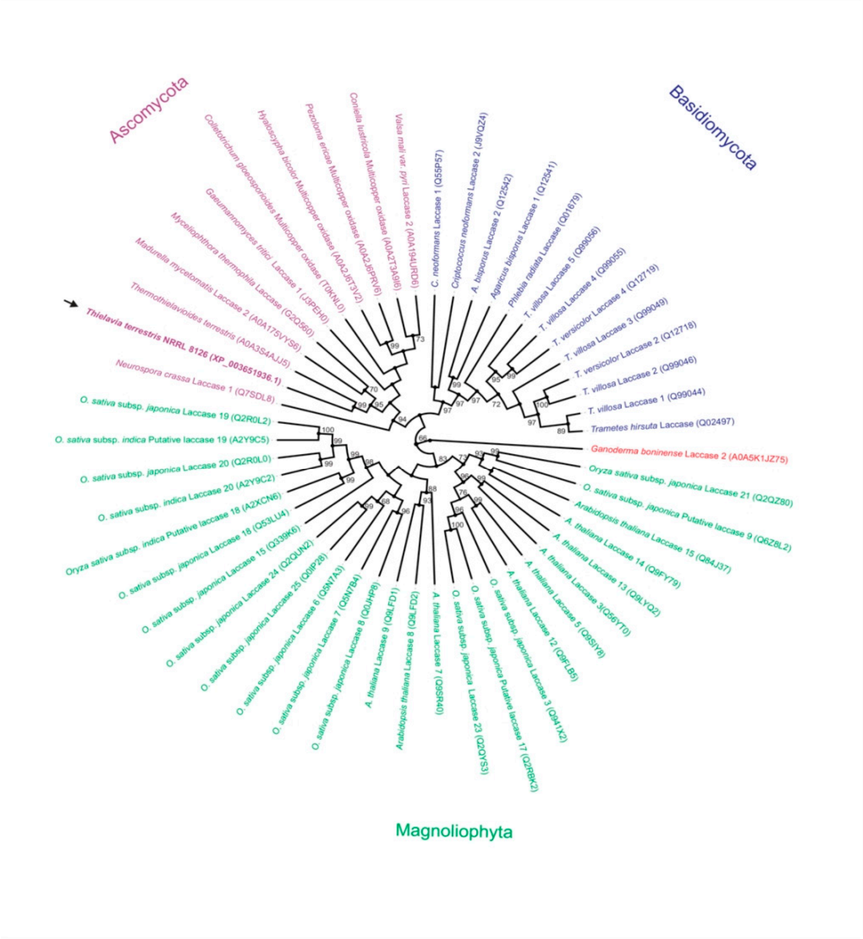

Figure S1. Maximum-likelihood tree (**2**), of multicopper oxidase from *T. terrestris* NRRL 8126 (UniProtKB: G2R0D5) with other multicopper oxidases and laccases. The analysis involved 52 amino acid sequences, accession numbers of NCBI database are shown in the branches of the tree. The model LG (-InL = -30529.08, G = 1.359, I = 0.006) was used for the analysis. *G. boninense* (UniProtKB: A0A5K1JZ75) was used as outgroup. The robustness at each node was assessed after 1000 pseudo-replicates and bootstrap support values are indicated for major nodes having 50 % values.
