# Supplementary material for: Improvement of Laccase Production by Thielavia terrestris Co3Bag1. Enhancing the Bio-Catalytic Performance of the Native Thermophilic TtLacA via Immobilization in Copper Alginate Gel Beads
Source: J Fungi (Basel). 2023 Feb 28;9(3):308. doi: 10.3390/jof9030308 (PMC10057929; doi:10.3390/jof9030308)
Supplement: Supplementary file 1 [file jof-09-00308-s001.zip › 3.Table S1 .pdf]

Table S1. Comparison biochemical properties of free and immobilized *Tt*LacA and others laccases

Strain/<br>Laccase name/substrate	MW<br>(kDa)	Support	Optimal Temperature (°C)		Thermal stability (Relative activity %)		Optimal pH		K_m (μM)		V_{max} (μMmin^{-1})		K_{cat} (s^{-1})		k_{cat}/K_m (s^{-1} μM^{-1})		Storage stability (Residual activity %/Time)		Reusability (Relative activity %/ Cycles reuse)	Reference
			Free	Imm	Free	Imm	Free	Imm	Free	Imm	Free	Imm	Free	Imm	Free	Imm	Free	Imm	Imm	
Chaetomium* sp. /-/ *	68	-	60	-	85 %,<br>50 °C, 90 min	-	3.0	-	10.2	-	-	-	-	-	-	-	-	-	-	[57]
G. australe*/Galacc-F/	48	-	55	-	-	-	6.0	-	164.137	-	-	-	273	-	1.663	-	-	-	-	[52]
G. luxurians*/GGL/	64	-	55-65	-	63 %,<br>50 °C, 60 min<br>52 %,<br>60 °C, 240 min	-	2.2	-	539	-	-	-	-	-	-0.140<br>140 (s^{-1} mM^{-1})		-	-	-	[59]
P. variabile*/PvL/	84	-	50	-	50 %,<br>50 °C, 60 min	-	4.8	-	203	-	40	-	-	-	-	-	80 / 1 day[b]	-	-	[51]
T. polyzona* WRF03/TpL/	66	-	65	-	42.92 %,<br>50 °C, 120 min	-	4.5	-	8.66	-	1429	-	-	-	-	-	-	-	-	[49]
T. harzianum* S7113/LacA/	63	-	50	-	50 %,<br>40 °C, 180 min	-	3.0	-	100<br>0.100 mM	-	0.603	-	-	-	-	-	-	-	-	[77]
T. trogii* S0301/Lac 37 II/	56	-	60	-	50 %,<br>60 °C, > 360 min<br>50 %,<br>70 °C, > 120 min	-	2.7	-	16.1	-	-	-	2.977	-	184.9	-	-	-	-	[67]
Thielavia* sp./-/*	70	-	70	-	-	-	5.0	-	23.7	-	-	-	4.14	-	0.1743	-	-	-	-	[48]
C. fabianii*/-/	52	calcium alginate gel beads<br>copper alginate gel beads	40	60<br>50	-<br>-	-<br>-	5.0	6.0<br>6.0	32<br>0.032 mM	78<br>0.078 mM<br>91<br>0.091 mM	0.015<br>15.0<br>mMmin^{-1}	0.00698<br>6.98 mMm^{-1}<br>0.00561<br>5.61 mMm^{-1}	-	-<br>-	-	-<br>-	62 / 21 days[b]	74 / 21 days[b]<br>67 / 21 days[b]	55/ 4[a]<br>60 / 4[a]	[10]
M. thermophila*/-/	-	carbon nanotube membrane	70	70	26.66 %,<br>50 °C, 240 min<br>1.86 %,<br>60 °C, 240 min	72.93 %,<br>50 °C, 240 min<br>23.12 %,<br>50 °C, 240 min	3.0	3.0	-	-	-	-	-	-	-	-	-	-	95 / 10<br>(25 °C, pH 4.5)	[61]
T. pubescens*/Tplac/	68	chitosan beads	50	60	-	-	4.5	5.0	-	-	-	-	-	-	-	-	15 / 30 days[a]	40 / 30 days[a]	≥ 60 / 6[a]	[50]

T. versicolor*/_/*	-	copper alginate gel beads	-	-	-	-	-	-	2210 2.21 mM	560 0.56 mM	0.00543 5.43 $mMmin^{-1}$	0.04464 44.64 $mMmin^{-1}$	-	-	-	-	46.18/ 15 days[b] (pH 5)	66.19 / 15 days[b] (pH 5)	21.5 / 5 NR	[81]
Bacillus* sp. MSK-01/-/	-	copper alginate gel beads	85	75	50 %, 70 °C, inactive	50 %, 70 °C, 240 min	8.0	10	-	-	-	-	-	-	-	-	-	> 90 / 15 days[b]	100 / 4 (75 °C, pH 8)	[26]
T. harzianum* HZN10/-/		calcium alginate gel beads		50	-	-		5.0		-	-	-	-	-	-	-		75/8 days[b]	36/ 6[a]	
	-	copper alginate gel beads	50	50	-	-	5.0	5-6	500 0.5 mM	-	-	-	-	-	-	-	45/8 days[b]	33 /8 days[b]	51/ 6[a]	[60]
		sol gel		50	-	-		4-7		-	285 U/mg	500 U/mg	-	-	-	-		90 /8 days[b]	82 /6[a]	
T. terrestris* Co3Bag1/*Tt*LacA/	70	copper alginate gel beads	65	70	50 %, 60 °C, 82.9 min 50 %, 70 °C, 50.3 min	50 %, 60 °C, 191.5 min 50 %, 70 °C, 117.2 min	3.0	3.0	260	450	3.57 U/mg	2.86 U/mg	13.73	6.35	0.053	0.0141	36.4/12 days[b] (pH 3)	86.1/12 days[b] (pH 3)	95 / six cycles (60 °C, pH 3)	This study

Imm:immobilized enzyme
* ABTS substrate; ** 2,6-DMP substrate
(a) optimal temperature and pH
(b) 4 °C
NR: No registered
